# GDNF Receptor Agonist Alleviates Motor Imbalance in Unilateral 6-Hydroxydopamine Model of Parkinson’s Disease

**Published:** 2020-11-24

**Authors:** Juho-Matti Renko, Merja H. Voutilainen, Tanel Visnapuu, Yulia A. Sidorova, Mart Saarma, Raimo K. Tuominen

**Affiliations:** 1 Division of Pharmacology and Pharmacotherapy, University of Helsinki, Finland; 2 Laboratory of Molecular Neuroscience, Institute of Biotechnology, University of Helsinki, Finland

**Keywords:** dopamine neurons, Glial cell line-Derived Neurotrophic Factor (GDNF), Parkinson’s disease, receptor tyrosine kinase RET agonist, unilateral 6-hydroxydopamine (6-OHDA) model

## Abstract

Parkinson’s disease (PD) is an incurable neurodegenerative disorder affecting up to 10 million people in the world. Diagnostic motor symptoms of PD appear as a result of progressive degeneration and death of nigrostriatal dopamine neurons. Current PD treatments only relieve symptoms without halting the progression of the disease, and their use is complicated by severe adverse effects emerging as the disease progresses. Therefore, there is an urgent need for new therapies for PD management. We developed a small molecule compound, BT13, targeting receptor tyrosine kinase RET. RET is the signalling receptor for a known survival factor for dopamine neurons called glial cell line-derived neurotrophic factor (GDNF). Previously we showed that BT13 prevents the death of cultured dopamine neurons, stimulates dopamine release and activates pro-survival signalling cascades in naïve rodent brain. In the present study, we evaluate the effects of BT13 on motor imbalance and nigrostriatal dopamine neurons in a unilateral 6-hydroxydopamine rat model of PD. We show that BT13 alleviates motor dysfunction in experimental animals. Further studies are needed to make a conclusion whether BT13 can protect the integrity of the nigrostriatal dopamine system since even the positive control, GDNF protein, was unable to produce a clear neuroprotective effect in the model used in the present work. In contrast to GDNF, BT13 is able to cross the blood-brain barrier, which together with the ability to reduce motor symptoms of the disease makes it a valuable lead for further development as a potential disease-modifying agent to treat PD.

## Introduction

Motor symptoms of Parkinson's disease (PD), the second most common neurodegenerative disorder, appear as a result of progressive degeneration of dopamine neurons in the substantia nigra pars compacta (SNpc) and their terminals in the putamen [1,2]. PD patients also suffer from a variety of non-motor symptoms [2–4] which are caused by the degeneration of different neuronal populations in the body. Current strategies for PD treatment are focused on dopamine supplementation, activation of dopamine receptors with small molecule agonists and prevention of dopamine degradation. Although these treatments improve the motor performance of PD patients, they fail to modify the course of the disease and are unable to protect or restore affected neurons. Available drugs also produce significant adverse effects and are unable to alleviate the non-motor symptoms. Therefore, novel treatments preferably with disease-modifying potential and ability to target non-motor symptoms are highly anticipated by PD patients.

Neurotrophic factors show robust neuroprotective effects, and therefore their receptors are appealing drug targets for the development of curative treatments for PD. In particular, glial cell line-derived neurotrophic factor (GDNF) and the related proteins forming together GDNF family ligands (GFLs) are well-known survival factors for nigrostriatal dopamine neurons [5–7]. In animal models of PD, GFLs alleviate motor symptoms, protect dopamine neurons and restore their function [5,6,8–12]. Until today, however, the clinical translation of GFLs has achieved only limited success. Although GFLs seemed to provide clinical benefits in a subset of PD patients [13–17], none of the randomised placebo-controlled Phase II trials with either GDNF protein or adeno-associated virus vector-encoded neurturin (AAV2-NRTN) met their primary endpoints [16–21]. Poor efficacy of GFLs in clinical trials can be attributed to their inability to cross the blood-brain barrier and limited diffusion in the brain tissue due to the high-affinity binding to extracellular matrix [22,23]. Indeed, the results of recent analysis of postmortem brain samples from PD patients treated with AAV2-NRTN show that upon the injection to the putamen NRTN immunoreactivity can be detected only in a portion of the putamen [24].

GFLs have to be delivered intracranially via a catheter surgically implanted into the brain. However, because of ethical reasons brain surgery is not recommended for PD patients at an early stage of the disease. Therefore, mainly patients with moderate or advanced PD were enrolled in clinical trials with GFLs. PD is a progressive disorder where the majority of dopamine neurons in the basal ganglia die within 5 years post diagnosis [25–27] making GFL-based treatments inefficient, because their target cells have already disappeared from the brain. In line with this concept, it was shown that the early-stage PD patients responded better to AAV2-NRTN therapy as compared to the late-stage patients [15].

We developed a small chemical compound targeting receptor tyrosine kinase RET, the major signalling receptor of GFLs. We named this compound BT13 and showed that it supports the survival of cultured midbrain dopamine neurons, stimulates dopamine release in the brain and activates pro-survival signalling cascades in the mouse striatum, thus mimicking the biological effects of GFLs in dopamine system [28]. In contrast to GDNF, BT13 readily distributes into tissues (volume of distribution >20 l/kg) and penetrates the blood-brain barrier (>50 % of the plasma concentration is found in the brain after an intravenous injection) [28,29]. Therefore, it can represent a better alternative for clinical translation as compared to GFL proteins.

Here, we tested the effects of BT13 on amphetamine-induced rotational behaviour in a unilateral 6-hydroxydopamine (6-OHDA) rat model of PD. The neuroprotective potential of the compound was evaluated postmortem by counting the number of cells expressing tyrosine hydroxylase (TH) in the SNpc and measuring the density of TH-positive and dopamine transporter (DAT)-positive fibers in the striatum. In this model, BT13 similarly to GDNF reduced motor imbalance of the experimental animals. Neither of the treatments significantly affected the density of TH-positive fibers in the striatum or the number of TH-positive cells in the SNpc. The density of DAT-positive fibers was higher in the striata of rats treated with GDNF. Our results indicate that BT13 is a weak RET agonist with GFL-like effects. Improvement of its intrinsic efficacy and physicochemical properties may result in the establishment of a new class of drugs with neuroprotective activity to treat early-phase PD.

## Materials and Methods

### Protein

Human recombinant hGDNF (produced in *E. coli*) was purchased from PeproTech (USA).

### BT13

The synthesis of BT13 was performed by EvoBlocks Ltd (Hungary). The purity of tested compound was 98.6% [28].

### Experimental animals

Adult male Wistar rats (RccHan:WIST, Harlan, the Netherlands), weighing 240–435 grams at the start of the experiments, were used for *in vivo* testing of BT13. All experiments were carried out according to the European Community guidelines for the use of experimental animals and approved by the National Animal Experiment Board of Finland (license numbers: ESAVI/7551/04.10.07/2013 and ESAVI/198/04.10.07/2014).

### 6-Hydroxydopamine lesion

Catecholaminergic neurotoxin 6-OHDA was injected unilaterally into the left dorsal striatum (AP=+1.0, ML=+2.7, DV=-4.0 mm relative to the bregma, according to the rat brain atlas [30] of male Wistar rats in a stereotaxic surgery under isoflurane (Vetflurane® 1000 mg/g, Virbac, France) anaesthesia [31]. Rats were fixed on a stereotaxic frame (Stoelting, USA) using ear bars and incisor bar. Lidocaine-adrenalin-solution (10 mg/ml; Orion Pharma, Finland) was injected between the skull and the scalp for local anaesthesia and to prevent bleeding. The skull was exposed, and a burr hole was made using a high-speed drill. An electronic injector (Quintessential stereotactic injector, Stoelting) and a 10 μl microsyringe (Hamilton Company, USA) were used to deliver 16 μg of 6-OHDA (Sigma-Aldrich, Germany), calculated as free base and dissolved in ice-cold saline with 0.02% ascorbic acid. Injection rate was set to 1.0 μl/min and injection volume to 4 μl. At the completion of the injection, the needle was kept in place for 4 minutes and then slowly withdrawn. Desipramine (15 mg/kg i.p.; calculated as free base; Sigma-Aldrich) was administered 30 minutes before the 6-OHDA injection to prevent the uptake of 6-OHDA into noradrenergic and serotonergic nerve terminals.

### Administration of BT13 and GDNF

Both BT13 and GDNF were delivered intracranially to directly compare their effects. For this purpose, another stereotaxic surgery was performed 1 hour after the 6-OHDA injection. The rats were anesthetized in the same manner as described above and the skull was exposed. A brain infusion cannula was implanted into the left dorsal striatum using the same coordinates as for the 6-OHDA injection (AP=+1.0; ML=+2.7; DV=-4.0 mm) and secured to the skull with 3 stainless steel screws and dental cement (Aqualox, Germany). The cannula was connected via a 3 cm-long catheter tubing to an osmotic infusion pump (Alzet model 2002, Durect Co., USA) which was placed into a subcutaneous pocket between scapulae. The pumps constantly infused BT13 (0.25 or 0.5 μg/μl in 100% propylene glycol), GDNF (0.25 μg/μl in PBS), 100% propylene glycol (PG) or PBS into the striatum at a flow rate of 0.5 μl/h for 7 days. At the end of the infusion, the pump, cannula, dental cement and screws were removed under isoflurane anaesthesia and the incision was cleaned and sutured.

Before the stereotaxic surgeries rats received buprenorphine 0.05 mg/kg s.c. (Temgesic® 0.3 mg/ml, Indivior UK Limited, UK) for analgesia. Carprofen 5 mg/kg (Rimadyl Vet® 50mg/ml, Zoetis Inc., USA) was injected s.c. immediately after the surgeries to relieve postoperative pain. Additional doses of buprenorphine and carprofen were given 1 day after the surgeries.

### Rotational asymmetry

Amphetamine-induced rotational behaviour was measured 2, 4 and 6 weeks after the 6-OHDA lesion in automatic rotometer bowls (Med Associates Inc., USA) as described previously [32,33]. After a habituation period of 30 minutes, rats received a single injection of D-amphetamine (2.5 mg/kg i.p.; calculated as free base; Division of Pharmaceutical Chemistry and Technology, University of Helsinki, Finland). Automated rotation sensors recorded full (360°) uninterrupted turns for a period of 120 minutes. Net ipsilateral turns to the lesion side were calculated by subtracting contralateral turns from ipsilateral turns. After completion of the last behavioral test, rats were anesthetized with sodium pentobarbital (90 mg/kg, i.p.; Mebunat Vet® 60 mg/ml, Orion Pharma) and perfused transcardially first with PBS for 5 minutes followed by 4% PFA in PBS for 10 minutes. The brains were excised, post-fixed in 4% PFA overnight at 4°C and stored in 20% sucrose in PBS at 4°C until snap-freezing in dry ice-cooled isopentane.

### Immunohistochemistry

To assess the number of remaining dopamine neurons in the SNpc and the density of dopaminergic fibers in the striatum, the brains were cut into 40-μm thick free-floating coronal cryosections. Endogeneous peroxidase activity was quenched in 3% H_2_O_2_ solution for 15 minutes. For DAT staining, antigen retrieval was performed by incubating the sections in 10 mM citrate buffer (pH 6) for 30 minutes at 80°C. The sections were then probed with monoclonal mouse anti-TH antibody (1:2000, Millipore Cat# MAB318, RRID:AB_2201528) or with monoclonal rat anti-DAT antibody (1:2000, Millipore Cat# MAB369, RRID:AB_2190413) overnight as described previously [31,34]. After rinsing in PBS, for TH staining the sections were incubated for 2 hours at room temperature in biotinylated horse anti-mouse secondary antibody solution (1:200, Vector Laboratories Cat# BA-2001, RRID:AB_2336180), and for DAT staining for 1 hour at room temperature in biotinylated rabbit anti-rat secondary antibody solution (1:200, Vector Laboratories Cat# BA-4000, RRID:AB_2336206). Thereafter, immunolabelling was reinforced with avidin-biotinylated HRP complex (Vectastain Elite ABC HRP Kit, Vector Laboratories Cat# PK-6100, RRID:AB_2336819), and visualized using DAB (0.5 mg/ml in 0.03% H_2_O_2_ in PBS; Cat# SK-4100, USA) as a chromogen. The stained sections were dehydrated in series of ethanol solutions with increasing concentrations, clarified in xylen and finally mounted in DePeX® mounting medium (VWR International Ltd., UK).

### Stereological assessment of TH-positive cells in the SNpc

TH-positive cell bodies in the SNpc were counted by a person blinded to the treatment groups using Olympus BX51 (Olympus Corporation, Japan) microscope connected to a computer running Stereo Investigator software version 11.06.2 (MBF Bioscience, USA) as described previously [31,33]. Optical fractionator probe with systematic random sampling was used for cell counting in Stereo Investigator and the parameters were set to the following values in each case: grid size: 92 × 92 μm; counting frame size: 80 × 80 μm; dissector height: 13 μm; guard zones: 1.5 μm at the bottom and top of section.

SNpc was outlined with a contour under 4X magnification (Olympus UPlanFl 4X/0.13 objective) and the subsequent cell counting was done under 60X magnification (Olympus PlanApo 60X 1.40 Oil ∞/0.17 objective) using immersion oil. TH-positive cell bodies were bilaterally counted in the SNpc on 3 coronal sections at approximately the same rostro-caudal levels, which were visually identified with the following landmarks: (1) medial terminal nucleus of the accessory optic tract (MT) partially divides the SNpc, (2) MT divides the SNpc into two parts, and (3) clusters of blood vessels enter the midbrain from the ventral side towards the SN without MT being present on that section. The counting contour always excluded the lateral part of the SNpc. In the case of (1) and (2) the medial edge of the SNpc was limited by the MT. In the case of (3), approximately equal distance from the lateral edge of the SNpc was taken to the medial direction on both sides with the aim to count only the SNpc and to exclude counting of the ventral tegmental area cells. Cells needed to have at least one long neurite and be polygonal to be included in the counting. The results are expressed as percentage of estimated number of TH-positive cells on the lesioned side relative to the intact side.

### Assessment of TH- and DAT-positive fiber density in the striatum

Analysis of TH- and DAT-positive fibers in the striatum was performed under blinded conditions. The density of TH- and DAT-positive fibers in the dorsal striatum was measured bilaterally from 3 coronal sections of each rat at the approximate rostro-caudal levels of AP=+1.2; +0.5 and -0.3 mm, relative to the bregma, according to the rat brain atlas [30]. Digital images of the immunolabelled sections were acquired with an automated bright field microscopy slide scanner (3DHistech Ltd., Hungary). Obtained images were converted to 8-bit grey scale, after which the colours were inverted and the integrated optical densities of the dorsal striata divided by the measured area were analyzed with Fiji ImageJ software (Media Cybernetics Inc, USA). Nonspecific background staining was measured from corpus callosum and subtracted from the striatal optical densities. The data are presented as percentage of the lesioned striatum as compared to the intact striatum.

### Statistical analysis

The number of animals per group was selected on the basis of our previous experience [31,33]. The data were subjected to statistical analysis using Student's *t*-test or one-way ANOVA followed by Tukey HSD *post hoc* tests in SPSS® Statistics 22 (IBM SPSS Inc., USA) software. Data were excluded from the analysis if they exceeded Mean ± 2X Standard Deviation. Vehicle-treated rats had to rotate more than 50 net ipsilateral turns in 120 minutes at 2 weeks post lesion to be included in the analysis. Results are presented as Mean ± SEM and considered statistically significant at *p*<0.05.

## Results

### BT13 alleviates motor deficit in 6-OHDA rat model of Parkinson’s disease

**Figure 1:**
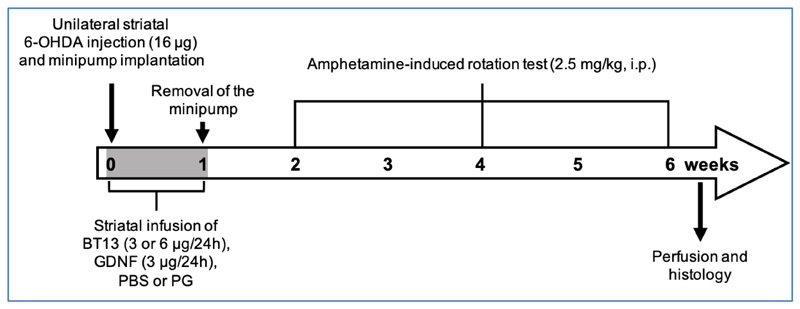
Scheme of the *in vivo* neuroprotection experiment.

**Figure 2:**
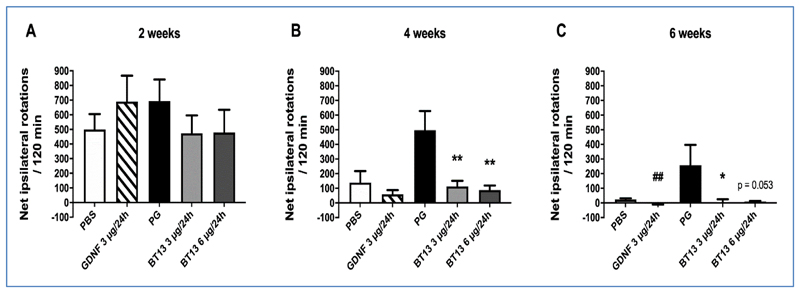
Amphetamine-induced turning behaviour is normalized by BT13 and GDNF in a time-dependent manner. The number of amphetamine-induced rotations at 2 weeks (A), 4 weeks (B), and 6 weeks post lesion (C). PG - propylene glycol (vehicle for BT13). *p<0.05, **p<0.01 as compared to PG, one-way ANOVA with Tukey HSD *post hoc* test, ## *p*<0.01 as compared to PBS, Student's unpaired *t*-test; Mean ± SEM, PBS N=6, GDNF 3 μg/24h N = 12-13, PG N=7-8, BT13 3 μg/24h N = 10-11, BT13 6 μg/24h N=8-9.

We compared the effects of BT13 and GDNF on motor deficits in a unilateral 6-OHDA model of PD in rats using neuroprotective paradigm where the drugs were delivered almost immediately after the lesion. The scheme of the experiment is presented in Figure 1. In vehicle treated rats, amphetamine induced strong ipsilateral turning behaviour at 2 weeks post lesion (499.2 ± 105.3 and 692.5 ± 148.8 net ipsilateral turns in PBS group and PG group, respectively) indicating unilateral damage of the dopaminergic system on the 6-OHDA lesioned side of the brain (Figure 2A). The number of net ipsilateral turns was in the same range in all treatment groups at 2 weeks post lesion. At 4 weeks post lesion, the number of net ipsilateral turns in animals treated with both BT13 3 μg/24h (112.6 ± 37.8) and BT13 6 μg/24h (87.5 ± 31.5) was significantly reduced when compared to the corresponding PG-vehicle (496.8 ± 131.3; *p*<0.01, Tukey HSD after one-way ANOVA) (Figure 2B). There was no statistically significant difference in the number of net ipsilateral turns between GDNF 3 μg/24 h (58.8 ± 28.2) and PBS (138.8 ± 78.4; *p*=0.21, Student's unpaired *t*-test)-treated rats.

At 6 weeks post lesion, both GDNF 3 μg/24 h and BT13 at the dose 3 μg/24 h produced a profound and comparable improvement in the turning behaviour (Figure 2C). GDNF-infused rats (-5.6 ± 5.6 net ipsilateral turns) rotated significantly less as compared to PBS-infused controls (23.3 ± 8.2 net ipsilateral turns; *p*=0.0064, Student's unpaired *t*-test). The number of net ipsilateral turns was significantly smaller in BT13 3 μg/24h -treated animals (3.7 ± 20.9) than in PG-treated animals (257.3 ± 138.8; *p*<0.05, Tukey HSD after one-way ANOVA). There was also a clear tendency towards reduction in the number of ipsilateral turns in animals treated with BT13 at the dose 6 μg/24h (9.6 ± 3.2; *p*=0.053, Tukey HSD after one-way ANOVA).

Noteworthily, in line with previously published data on this particular toxin administration scheme [35], we observed spontaneous recovery in the turning behaviour especially in PBS-treated rats whose net ipsilateral turns reduced from 499.2 ± 105.3 at 2 weeks post lesion to 138.8 ± 78.4 and 23.3 ± 8.2 at 4 and 6 weeks post lesion, respectively.

### Effect of BT13 on TH-immunoreactive neurons in the SNpc and TH- and DAT-immunoreactive fibers in the striatum of 6-OHDA lesioned rats

**Figure 3:**
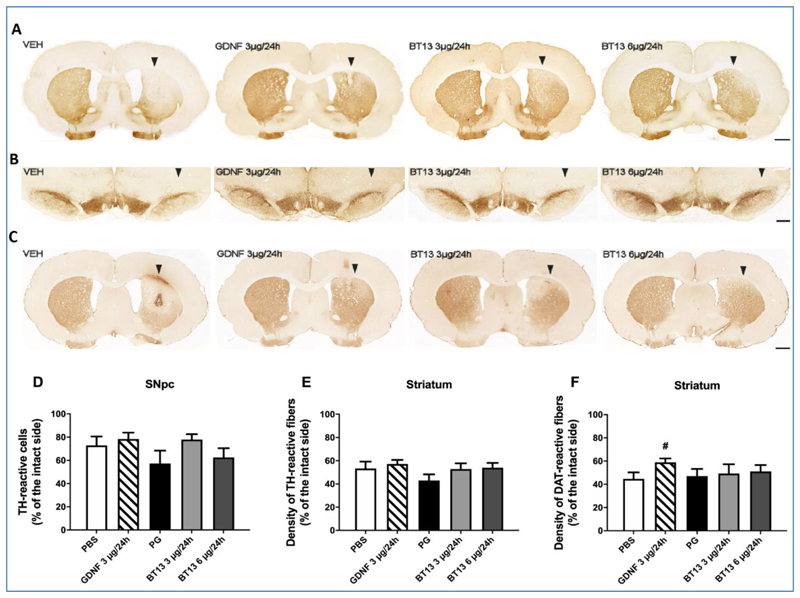
Effect of BT13 and GDNF on the density of TH and DAT-immunoreactive fibers in the dorsal striatum and the number of TH-immunoreactive cells in the SNpc in 6-OHDA lesioned rats. Representative images of TH immunohistochemical staining in the striatum (A), and in the central SNpc (B), and DAT immunoreactivity in the striatum (C) in different treatment groups at 6 weeks post lesion. (D) Quantification of the number of TH-immunoreactive cells in the SNpc. (E) Quantification of the density of TH-immunoreactive fibers in the dorsal striatum. (F) Quantification of the density of DAT-immunoreactive fibers in the dorsal striatum. Scale bar in (A) and (C) is 1 mm and in (B) 0.5 mm. The lesion-side is denoted with arrowheads. # *p*<0.05 as compared to PBS, Student's unpaired *t*-test; Mean ± SEM. In TH staining (D,E) PBS N=6, GDNF 3 μg/24h N=12-13, PG N=8, BT13 3 μg/24h N=11-12, BT13 6 μg/24h N=8; in DAT staining (F) PBS N=6, GDNF 3 μg/24h N = 13, PG N=8, BT13 3 μg/24h N=11, BT13 6 μg/24h N=8. TH - tyrosine hydroxylase, DAT – dopamine transporter, PG – propylene glycol.

To assess the integrity of the nigrostriatal dopamine system in the neuroprotection study, coronal brain sections from the SNpc and striatum were analysed histologically after the last behavioural test. The single unilateral injection of 6-OHDA into the dorsal striatum resulted in a relatively mild degeneration of dopamine neurons: in rats treated with PBS and PG, the density of TH-positive fibers in the striatum was reduced by 46.8 ± 6.0% and 57.0 ± 5.3% in comparison to the intact side, respectively (Figures 3A, 3E). Unexpectedly GDNF infusion did not increase the density of TH-positive fibers in the lesioned striatum (42.8 ± 3.4% loss in TH-immunoreactivity) as compared to PBS (*p*=0.76; Student's unpaired *t*-test) (Figure 3E). Both of the tested doses of BT13, 3 μg/24h and 6 μg/24h, produced a slight increase in the density of TH-positive fibers (47.1 ± 4.8% and 46.0 ± 4.1% loss in TH-immunoreactivity, respectively) but this effect was not statistically significant when compared to the PG-vehicle. The number of TH-positive cell bodies in the SNpc on the 6-OHDA lesioned side was reduced by 27.2 ± 7.7% in PBS-treated rats and by 42.8 ± 11.2% in PG-treated rats (Figures 3B, 3D). Intrastriatal infusion of BT13 or GDNF was unable to significantly protect TH-positive cells in the SNpc although in rats treated with BT13 3 μg/24h (22.2 ± 4.7% loss of TH-immunoreactive cells), the number of surviving TH-positive cell bodies in the SNpc was approximately 20% higher than in rats treated with PG (Figure 3D). Since GDNF overexpression or injection into the striatum can downregulate TH expression [36–38], we analysed the density of DAT-immunoreactive fibers in the striatum of GDNF and BT13 treated rats (Figures 3C, 3F). The density of DAT-positive fibers was significantly higher in GDNF-treated rats (58.9 ± 3.4% DAT-immunoreactivity *vs.* intact side) than in PBS-treated rats (44.6 ± 5.6%; *p*=0.035, Student's unpaired *t*-test) (Figure 3F). Thus, it is possible that TH expression could have been slightly downregulated by GDNF in this study protocol.

## Discussion

In the present study, we demonstrated that a small molecule RET agonist, BT13, alleviates motor imbalance in a rat unilateral model of PD when compared with corresponding control (PG). We also attempted to evaluate the neuroprotective potential of this compound by assessing the integrity of the nigrostriatal dopamine pathway, but the results remained inconclusive. Neither BT13, nor GDNF, which was used as a positive control in this study, was able to significantly protect TH-positive fibers in the striatum or dopamine neurons in the SNpc of 6-OHDA lesioned rats. Nevertheless, in the striata of GDNF-treated rats we observed small but significant increase in the density of DAT-positive fibers. Taken together, these data suggest that GDNF likely downregulated the expression of TH after a seven-day striatal infusion as it has been shown by other research groups [36–38]. Whether BT13 is expected to produce a similar effect is unknown, but since both GDNF and the small molecule compound signal through the same receptors, it cannot be excluded [28].

GDNF is known to protect and restore nigrostriatal dopamine neurons against toxin-induced damage in animal models of PD and preserve normal motor functions in 6-OHDA lesioned animals [8,10]. In the present study, there was obvious spontaneous recovery in PBS-treated rats and therefore the neuroprotective effect of GDNF, dissolved in PBS, was rather difficult to observe. Spontaneous recovery was less prominent in PG-treated rats and BT13, which was dissolved in PG, was able to induce recovery of motor imbalance. According to our previously published results, the efficacy and potency of BT13 in integral assays are lower compared to those of GDNF [29]. Therefore, it is difficult to expect statistically significant improvement in response to this compound in a model where the effect of GDNF is barely seen. In general, the model we used in this study was characterized by a relatively mild lesion of dopamine neurons and significant spontaneous functional recovery.

Poor neuroprotective efficacy of either treatment in the animal model of PD used in this study can be also attributed to toxin-induced downregulation of GFL receptors. According to Marco et al. [39] 6-OHDA lesion downregulates the expression of RET and GFRα1 receptors in the rat striatum during the first week post-injection. As both GDNF and BT13 in this study were infused during the period of time when the receptors could have possibly been downregulated, we were not able to detect as prominent neuroprotective effect of BT13 and GNDF at the level of TH and DAT immunostainings as expected.

Another factor that could have contributed to the poor efficacy of GDNF in this model can be related to the use of PBS as a vehicle for the protein. PBS infusion to the brain will massively increase the activity of phosphatases. GDNF activates its receptor, the receptor tyrosine kinase RET by stimulation of phosphorylation. Phosphorylated tyrosine residues in RET trigger the activation of intracellular kinase cascades, such as MAPK and AKT [40]. Since the signalling of GFLs is heavily dependent on phosphorylation processes in cells, the activation of phosphatases [41] could have suppressed the effect of GDNF on dopamine neurons in the neuroprotection model.

It can also be speculated that the doses of BT13 and GDNF (or the biological activity of the particular batch of GDNF) were too low to elicit the protection of dopaminergic cell bodies in the SNpc after intrastriatal infusion. BT13 has limited solubility especially in aqueous solutions (≤ 100 μM). In the present study, we used PG to dissolve BT13. However, upon infusion into aqueous extracellular environment the low solubility of the compound could have led to partial precipitation and reduction of its free concentration in the brain. In addition, BT13 is metabolically unstable [29]. Limited solubility of BT13 also prevented us from testing higher doses of this compound in our experiments.

In contrast to GDNF, BT13 spreads well in tissues and is able to penetrate through tissue barriers [29]. Therefore, we cannot exclude the effects of BT13 on the expression of TH and DAT in the contralateral hemisphere. We express the density of TH- and DAT-immunoreactive fibers in percentage of the contralateral side. Thus, RET activation on the contralateral side resulting in enhanced axonal sprouting could have masked the neuroprotective effect of BT13 on dopamine neurons *in vivo.* Nevertheless, the infused concentration of BT13 was relatively low, and it is unclear if an effective dose of BT13 was able to reach the contralateral side of the brain.

In general, although BT13 did not show significant neuroprotection at the level of TH or DAT immunohistochemistry in this partial lesion model we saw a significant functional improvement in the behavioral tests (Figures 2A-2C). This may be i) due to prevention of toxin entry into dopamine neurons via influencing DAT activity by BT13, ii) due to dopamine mobilizing effect of BT13 as we showed previously [28], or iii) due to its neuroprotective effect on lesioned dopamine neurons. The first mentioned two options are, however, unlikely because BT13 was shown to mobilize dopamine after an acute administration whereas the amphetamine-induced turning behavior was reduced only at 4 weeks post lesion (i.e. 3 weeks after the cessation of the BT13 infusion). We also observed no changes in DAT activity in response to BT13 in an *in vitro* assay [29]. Thus, direct inhibition of 6-OHDA entry into neurons is improbable, although indirect interactions cannot be completely ruled out based on these results. BT13 similarly to GDNF can activate in the brain pro-survival signalling cascades, such as MAPK and AKT [28]. Therefore, it may have similar neuroprotective mechanism of action as GDNF in dopamine neurons, although further studies are needed to fully understand the effects of BT13 *in vivo.*

To summarize, the current results show that a small molecule agonist of GDNF receptor RET efficiently reduces motor deficit in an animal model of PD. This molecule is structurally distinct from the currently available PD medications and may give rise to the development of a novel class of therapeutics for PD management. However, BT13 should be further optimized to improve its physicochemical properties and biological activity. The clarification of its mode of action *in vivo* also requires additional experiments. Since it signals through the same receptors as GDNF, a protein with known neuroprotective and neurorestorative properties in nigrostriatal dopamine system, BT13 may not only alleviate the symptoms of PD patients, but also have disease-modifying properties.
